# Denosumab for the Treatment of Hypercalcemia in a Patient With Parathyroid Carcinoma: A Case Report

**DOI:** 10.3389/fendo.2021.794988

**Published:** 2022-01-31

**Authors:** Abdallah Roukain, Heba Alwan, Massimo Bongiovanni, Gerasimos P. Sykiotis, Peter A. Kopp

**Affiliations:** ^1^ Service of Endocrinology, Diabetes and Metabolism, Lausanne University Hospital, Lausanne, Switzerland; ^2^ Institute of Primary Health Care, University of Bern, Bern, Switzerland; ^3^ SYNLAB, Pathology and Cytopathology, Lausanne, Switzerland

**Keywords:** denosumab, hypercalcemia, parathyroid carcinoma, RANKL, calcimimetics

## Abstract

**Background:**

Refractory hypercalcemia is one of the major complications of parathyroid carcinoma.

**Case report:**

An 84-year old female patient presented with an acute confusional state due to hypercalcemia. This led to the diagnosis of primary hyperparathyroidism for which she underwent surgery. The initial histological diagnosis was interpreted as atypical parathyroid adenoma; the resection was microscopically incomplete. One year later, the patient presented with elevated calcium levels up to 3.89 mmol/l. Recurrent severe hypercalcemia required multiple hospitalizations. Review of the histology slides revealed that the initially resected lesion was in fact a parathyroid carcinoma. Treatment with the calcimimetic drug cinacalcet was poorly tolerated. Repeated administration of zoledronic acid only had transient effects on calcium levels, and bisphosphonate treatment was ultimately discontinued because of chronic renal failure. The patient then received denosumab (60 or 120 mg) when needed (nine doses over twenty months), the last dose in November 2020, which led to a reduction and control of here calcium levels. Currently, at three years after initial surgery, calcium levels are stable between 2.7-2.8 mmol/l and the patient has not required hospitalization for hypercalcemia for 10 months.

**Discussion:**

In case of parathyroid carcinoma, *en-bloc* resection is the first treatment. Denosumab has proven its efficiency in treating hypercalcemia in malignancy. Several case reports studied denosumab in hypercalcemia due to parathyroid carcinoma, and the treatment were efficient to decrease levels of calcium when repeated as needed or monthly. We report another case of refractory hypercalcemia treated with several doses of denosumab in a patient with parathyroid carcinoma.

## Introduction

Hypercalcemia due to primary hyperparathyroidism (PHPT) is a frequent pathology encountered by endocrinologists. The vast majority (>99%) of PHPT is caused by parathyroid adenomas, whereas parathyroid carcinomas are extremely rare (1, 2). Calcium and parathyroid hormone (PTH) levels are typically much higher in cases of parathyroid carcinomas compared to adenomas (3). The first case of parathyroid cancer was described by De Quervain et al. in 1909 (4).

Parathyroid carcinoma should be suspected when levels of PTH are high, in cases of severe hypercalcemia, and/or recurrent hypercalcemia. However, in some instances, the diagnosis of parathyroid carcinoma is not considered before surgery and the entity is only recognized retrospectively after surgical resection (1). Typically, hypercalcemia has a larger impact on morbidity and mortality than locoregional effects or the rare metastases of the tumor (5). If a parathyroid carcinoma is suspected, the first-line treatment consists of surgical *en bloc* resection together with the ipsilateral thyroid lobe, even in the presence of metastatic disease (1, 3). If the tumor is unresectable and/or hypercalcemia persists, medical therapy is necessary. In addition to appropriate hydration, the most commonly used agents include calcimimetics such as cinacalcet, or bisphosphonates (2, 3, 5). Calcitonin and octreotide can be used for hypercalcemic crises, but their effect is limited (1, 5). Although cinacalcet is now widely used in patients with persistent hypercalcemia, it is often poorly tolerated due to its gastrointestinal side effects, which are usually dose-dependent (1). Calcimimetics act through stimulation of the calcium-sensing receptor (CaSR), which results in a decrease of PTH synthesis and secretion (1). Bisphosphonates, through their inhibition of osteoclast-mediated bone resorption, tend to be helpful in controlling the hypercalcemia but their use is contraindicated in patients with impaired renal function (1). Other therapeutic modalities that have been proposed for the control of hypercalcemia associated with parathyroid carcinomas include chemotherapy (6, 7), radiation therapy (8, 9) [although parathyroid cancers are generally considered non-radiotherapy sensitive (1, 5)], and, more recently, denosumab (1).

Denosumab is a human monoclonal antibody to the receptor activator of NF-κB ligand (RANKL), which inhibits the maturation of osteoclasts. It is approved for the therapy of osteoporosis, as well as adjuvant therapy in patients with bone metastases of solid tumors (3, 10). Because of the rarity of parathyroid carcinomas, it is difficult to study larger case series, and reports on the use of denosumab for the control of refractory hypercalcemia in such patients are scarce (1, 3, 5, 11, 12).

We report an 84-year-old female patient who was found to have hypercalcemia due to PHPT after presenting with an acute confusional state. Her pre-operative albumin-corrected calcium (ACC) level was 3.6 mmol/l (reference range 2.2-2.6 mmol/l), and the PTH level was 293 ng/l.

## Clinical Presentation

**Figure 1:**
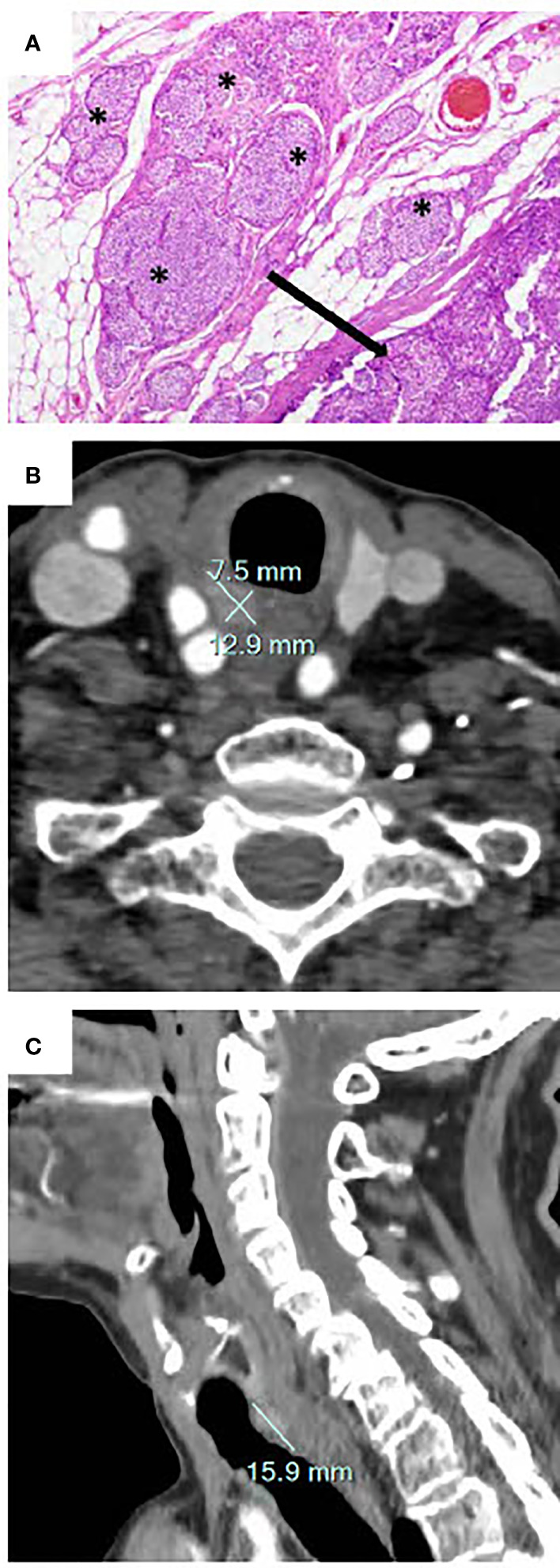
**(A)** Original histology showing the main mass (arrow) and tumoral nests (*) that extend into the adipose tissue. This invasion, together with vascular invasion, mitotic figures, and necrosis is indicative for a low-grade parathyroid carcinoma (hematoxylin & eosin staining, 40X). **(B, C)** Cervical computed tomography demonstrating a right retro-tracheal mass of 15.9 x 12.9 x 7.5 mm corresponding to local recurrence of the incompletely resected parathyroid carcinoma.

The histological diagnosis at the time was an atypical parathyroid adenoma; the presence of mitosis and extension beyond the parathyroid capsule were not considered as worrisome; resection was microscopically incomplete. Hypercalcemia resolved immediately post-operatively. Ten months after surgery, the ACC level was 2.6 mmol/l without treatment. In January 2019 (1 year after the surgery), the patient was hospitalized in our institution for symptomatic hypercalcemia, with an ACC level of 3.5 mmol/l and a concomitant PTH level of 724 ng/l (reference range 10-70 ng/l). The patient was treated with intravenous saline infusion, calcitonin, and an intravenous bisphosphonate (zoledronic acid 5 mg). Review of the original surgical pathology at our institution led to the conclusion that the tumor was in fact a parathyroid carcinoma (**Figure 1A**). Cervical ultrasound did not show any residual mass suspicious of a parathyroid adenoma or carcinoma. Imaging with fluorocholine (^18^FDG) PET/CT revealed a right retro-tracheal mass suggestive for residual/recurrent local disease (**Figures 1B, C**), a hypermetabolic left cervical lymph node that ultimately showed a benign cytology, and no evidence for distant metastasis. Targeted external radiotherapy (48 Gy) was delivered to the retropharyngeal mass.

Three months after external radiotherapy, cross-sectional imaging with CT and MRI showed that the retrotracheal mass was stable in size, in close proximity to the trachea, the esophagus, and the common carotid artery. Tracheal endoscopy did not show overt invasion of the trachea but the tumor exerted a mass effect on the trachea. The benefits and risks associated with a surgical resection of the mass were discussed in a multidisciplinary tumor board (endocrinologists, oncologists and otolaryngologists) and with the patient. Considering her advanced age and the expected morbidity including the potential need for a tracheostomy, the patient opted against surgical intervention and for continuing supportive care.

**Figure 2:**
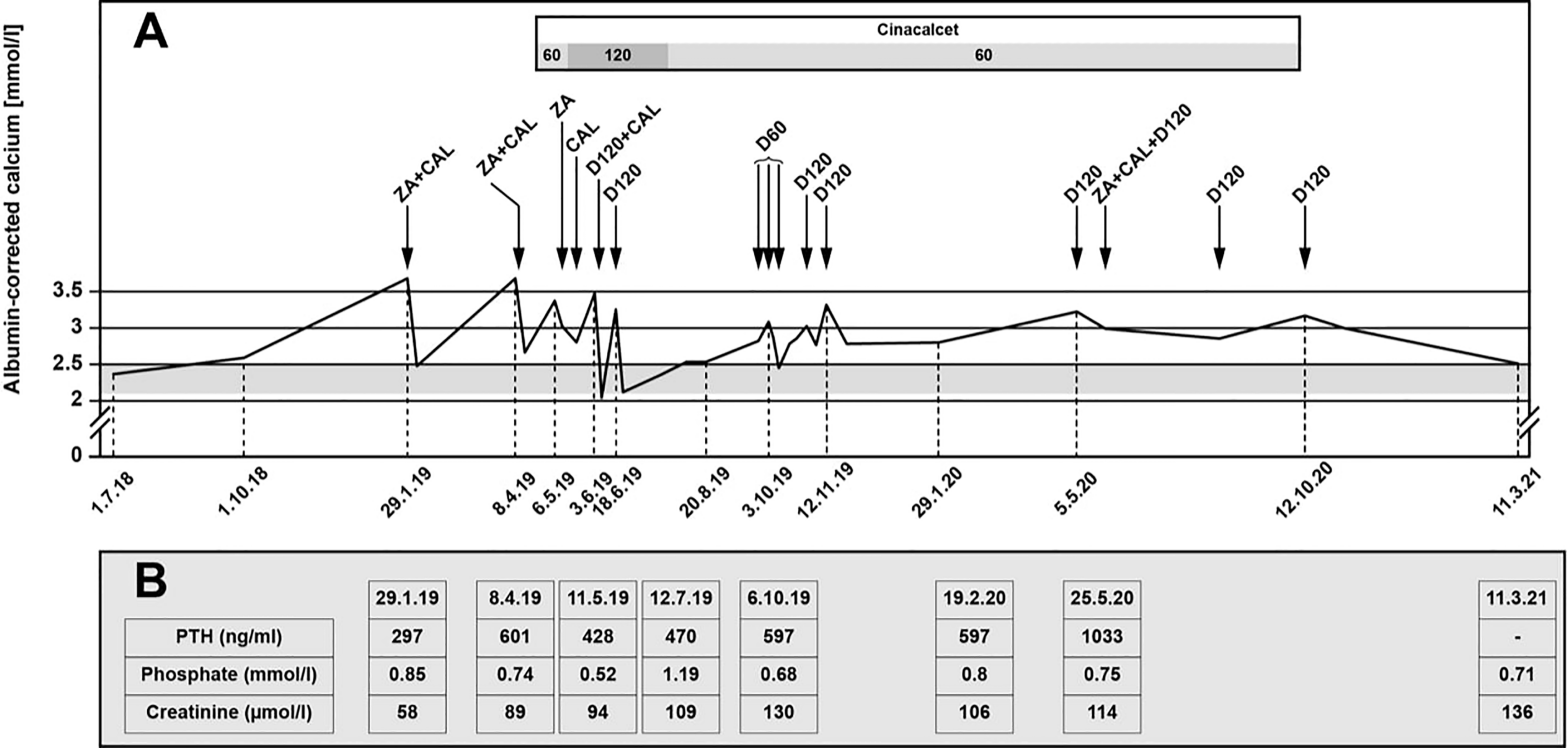
**(A)** Calcium levels under therapy with denosumab. ZA, zoledronic acid; CAL, calcitonin; D60, denosumab 60 mg; D120, denosumab 120. **(B)** Selected PTH, phosphate and creatinine levels. PTH, parathyroid hormone.

Despite adequate hydration and bisphosphonate therapy, the ACC remained high (up to 3.9 mmol/l). The patient was very symptomatic with severe fatigue, nausea and vomiting, and she required recurrent hospitalizations over a period of 2 months. Treatment with cinacalcet with increasing doses up to 90 mg twice daily was introduced, but the therapy was poorly tolerated, with worsening nausea and vomiting despite improvement of her ACC levels (2.5-2.6 mmol/l) and the use of multiple anti-emetic drugs (ondansetron, metoclopramide, prednisone). Because of these adverse effects, cinacalcet was reduced to 30 mg twice daily. This, however, resulted in worsening of her hypercalcemia and led again to recurrent hospitalizations. Although denosumab is not officially approved for the control of hypercalcemia in Switzerland, it was introduced with an initial dose of 120 mg, followed by eight doses of 60 or 120 mg (**Figure 2A**). Denosumab was administered at variable intervals between one to twelve weeks every time the ACC was > 3 mmol/l. Therapy with cinacalcet was discontinued by the patient because of nausea and vomiting.

Currently, at more than three years after initial surgery, calcium levels remain stable between 2.5-2.6 mmol/l and the patient has not required hospitalization for hypercalcemia in 10 months.

Selected additional biochemical markers such as phosphate levels are summarized in **Figure 2B**. The patient only developed mild hypophosphatemia, without further symptoms, which was corrected with oral phosphate replacement until the levels normalized. The administration of denosumab did not further impact the serum phosphate levels. PTH levels increased during follow-up. Creatinine levels fluctuated with several episodes with acute renal insufficiency associated with dehydration in the context of severe nausea and vomiting secondary to the hypercalcemia and side effects of cinacalcet.

## Discussion

Denosumab is commonly used for the treatment of osteoporosis and has proven efficacy in the treatment of hypercalcemia of malignancy. Denosumab was shown to be superior to zoledronic acid in patients with hypercalcemia due to bone metastasis in patients with prostate cancer (13). Hu et al. (14) used denosumab for hypercalcemia in different malignancies (solid and hematological) and the results showed a decrease of calcium levels in 64% of patients within 10 days (14). A meta-analysis of six randomized controlled trials (7772 patients included) investigated the efficacy of denosumab versus zoledronic acid in terms of “skeletal-related events”. The risk of hypocalcemia was higher in the denosumab group than the zoledronic acid group (relative risk 1.99, p = 0.02) (15).

If a parathyroid carcinoma is suspected, the initial management consists of *en bloc* resection of the tumor. In case of persistent hypercalcemia after initial surgery, and residual or recurrent tumor that is not amenable to surgical excision, the first-line treatment typically consists in the administration of a calcimimetic drugs such as cinacalcet, or/and the administration of bisphosphonates. Although not approved for this indication, denosumab has been successfully used in a small number of patients with refractory hypercalcemia caused by unresectable parathyroid carcinomas (1–3, 5, 11, 12). To date, there are no recommendations for the use of denosumab in terms of timing and doses. Some authors have administered it once per month (2, 5), but others (3), including our group, administered denosumab whenever the calcium levels rose to levels > 3 mmol/l, a level that was associated with clear clinical signs of hypercalcemia. It is also recommended to monitor serum phosphate levels after administration of denosumab as hypophosphatemia can occur in 16-32% of patients receiving this treatment (16). In agreement with others (1, 3, 5, 11, 12), we recommend that denosumab be considered for the treatment of refractory hypercalcemia in patients with parathyroid carcinomas.

## Conclusion

In refractory hypercalcemia due to parathyroid carcinoma resistant to treatment by calcimimetics and/or bisphosphonates, denosumab is an effective alternative that permits to effectively lower calcium levels, improve symptoms and quality of life, and avoid repeated hospitalizations.

## Author’s Note

The abstract of this work was presented in abstract form at the SSED (Swiss Society of Endocrinology and Diabetology) meeting 2020.

## Author Contributions

Author Contributions: writing—original draft preparation, AR and PK. Writing—review and editing, AR, HA, MB, GS, and PK. Supervision, PK. All authors have read and agreed to the published version of the manuscript.

## Conflict of Interest

The authors declare that the research was conducted in the absence of any commercial or financial relationships that could be construed as a potential conflict of interest.

## Publisher’s Note

All claims expressed in this article are solely those of the authors and do not necessarily represent those of their affiliated organizations, or those of the publisher, the editors and the reviewers. Any product that may be evaluated in this article, or claim that may be made by its manufacturer, is not guaranteed or endorsed by the publisher.

## References

[1] Betea D Potorac I Beckers A. Parathyroid Carcinoma: Challenges in Diagnosis and Treatment. Annales D’endocrinol (2015) 76(2):169–77. doi: 10.1016/j.ando.2015.03.003 25910997

[2] Bowyer SE White AM Ransom DT Davidson JA. Resistant Hypercalcaemia in Metastatic Parathyroid Carcinoma. Med J Aust (2013) 198(10):559–61. doi: 10.5694/mja12.11243 23725272

[3] Vellanki P Lange K Elaraj D Kopp PA El Muayed M. Denosumab for Management of Parathyroid Carcinoma-Mediated Hypercalcemia. J Clin Endocrinol Metab (2014) 99(2):387–90. doi: 10.1210/jc.2013-3031 PMC3913820 24178790

[4] de Quervain F. Parastruma Maligna Aberrata. Deutsche Z F Chirurgie (1909) 100(1):334–53. doi: 10.1007/BF02819737

[5] Karuppiah D Thanabalasingham G Shine B Wang LM Sadler GP Karavitaki N. Refractory Hypercalcaemia Secondary to Parathyroid Carcinoma: Response to High-Dose Denosumab. Eur J Endocrinol (2014) 171(1):K1–5. doi: 10.1530/EJE-14-0166 24743399

[6] Calandra DB Chejfec G Foy BK Lawrence AM Paloyan E. Parathyroid Carcinoma: Biochemical and Pathologic Response to DTIC. Surgery (1984) 96(6):1132–7. 6505966

[7] Bukowski RM Sheeler L Cunningham J Esselstyn C. Successful Combination Chemotherapy for Metastatic Parathyroid Carcinoma. Arch Internal Med (1984) 144(2):399–400. doi: 10.1001/archinte.1984.00350140229032 6696578

[8] Chow E Tsang RW Brierley JD Filice S. Parathyroid Carcinoma–The Princess Margaret Hospital Experience. Int J Radiat Oncol Biol Phys (1998) 41(3):569–72. doi: 10.1016/S0360-3016(98)00098-4 9635703

[9] Munson ND Foote RL Northcutt RC Tiegs RD Fitzpatrick LA Grant CS. Parathyroid Carcinoma: Is There a Role for Adjuvant Radiation Therapy? Cancer (2003) 98(11):2378–84. doi: 10.1002/cncr.11819 14635072

[10] Meier C Uebelhart B Aubry-Rozier B Birkhäuser M Bischoff-Ferrari HA Frey D. Osteoporosis Drug Treatment: Duration and Management After Discontinuation. A Position Statement From the SVGO/ASCO. Swiss Med Weekly (2017) 147:w14484. doi: 10.4414/smw.2017.14484 28871570

[11] Nadarasa K Theodoraki A Kurzawinski TR Carpenter R Bull J Chung TT. Denosumab for Management of Refractory Hypercalcaemia in Recurrent Parathyroid Carcinoma. Eur J Endocrinol (2014) 171(3):L7–8. doi: 10.1530/EJE-14-0482 24939719

[12] Jumpertz von Schwartzenberg R Elbelt U Ventz M Mai K Kienitz T Maurer L. Palliative Treatment of Uncontrollable Hypercalcemia Due to Parathyrotoxicosis: Denosumab as Rescue Therapy. Endocrinol Diabetes Metab Case Rep (2015) 2015:150082. doi: 10.1530/EDM-15-0082 26605043 PMC4653612

[13] Fizazi K Carducci M Smith M Damião R Brown J Karsh L. Denosumab Versus Zoledronic Acid for Treatment of Bone Metastases in Men With Castration-Resistant Prostate Cancer: A Randomised, Double-Blind Study. Lancet (Lond Engl) (2011) 377(9768):813–22. doi: 10.1016/S0140-6736(10)62344-6 PMC3090685 21353695

[14] Hu MI Glezerman IG Leboulleux S Insogna K Gucalp R Misiorowski W. Denosumab for Treatment of Hypercalcemia of Malignancy. J Clin Endocrinol Metab (2014) 99(9):3144–52. doi: 10.1210/jc.2014-1001 PMC4154084 24915117

[15] Menshawy A Mattar O Abdulkarim A Kasem S Nasreldin N Menshawy E. Denosumab Versus Bisphosphonates in Patients With Advanced Cancers-Related Bone Metastasis: Systematic Review and Meta-Analysis of Randomized Controlled Trials. Support Care Cancer (2018) 26(4):1029–38. doi: 10.1007/s00520-018-4060-1 29387997

[16] Megapanou E Florentin M Haralampos M Elisaf M Liamis G. Drug-Induced Hypophosphatemia: Current Insights. Drug Saf (2020) 43:197–210. doi: 10.1007/s40264-019-00888-1 31776845

